# Circulating Adipsin as a Biomarker of Liver Fat Content in Prepubertal Children Born Small-for-Gestational-Age

**DOI:** 10.3390/ijms27115023

**Published:** 2026-06-02

**Authors:** Marta Díaz, Marion Peyrou, Abel López-Bermejo, Francesc Villarroya, Francis de Zegher, Paula Casano-Sancho, Lourdes Ibáñez

**Affiliations:** 1 Endocrinology Department, Institut de Recerca Sant Joan de Déu, University of Barcelona, 08950 Barcelona, Spain; paula.casano@sjd.es; 2 Centro de Investigación Biomédica en Red de Diabetes y Enfermedades Metabólicas Asociadas (CIBERDEM), Instituto de Salud Carlos III, 28029 Madrid, Spain; 3 Biochemistry and Molecular Biomedicine Department, Institute of Biomedicine, University of Barcelona, 08007 Barcelona, Spain; marion.peyrou@ub.edu (M.P.); fvgombau@gmail.com (F.V.); 4 Network Biomedical Research Center of Physiopathology of Obesity and Nutrition (CIBEROBN), Instituto de Salud Carlos III, 28029 Madrid, Spain; 5 Institut de Recerca Sant Joan de Déu, Esplugues de Llobregat, 08950 Barcelona, Spain; 6 Pediatric Endocrinology Research Group, Girona Institute for Biomedical Research (IDIBGI), Faculty of Medicine, University of Girona and Dr. Josep Trueta Hospital, 17007 Girona, Spain; alopezbermejo@idibgi.org; 7 Leuven Research & Development, University of Leuven, 3000 Leuven, Belgium; francis.dezegher@kuleuven.be

**Keywords:** SGA, adipsin, body composition, liver fat

## Abstract

Adipsin is a serine protease secreted mainly by adipocytes with a key role in the regulation of lipid metabolism and energy homeostasis. Individuals born small-for-gestational-age (SGA) with excessive postnatal catch-up in weight are at risk of developing central (hepato-visceral) fat deposition and features of metabolic dysfunction-associated steatotic liver disease (MASLD). We assessed cross-sectionally the serum concentrations of adipsin in seventy-five prepubertal children, aged ~7.8 yr, born appropriate-for-gestational-age (AGA, N = 40) or SGA (with spontaneous catch-up, N = 35) and their association with markers of abdominal adiposity and metabolic health. Assessments included anthropometry, serum adipsin, glucose, insulin, high-molecular-weight adiponectin (HMW-adip), lipids, and abdominal fat partitioning by magnetic resonance imaging (MRI). SGA children had higher serum adipsin levels [1.9 mg/L ± 0.4 vs. 1.4 mg/L ± 0.1 (mean ± SEM); *p* < 0.001], a less favorable endocrine–metabolic profile, and more hepato-visceral fat. Adipsin concentrations correlated inversely with birth weight and HMW-adip concentrations and positively with markers of insulin resistance, abdominal adiposity, and with the percentage of liver fat. Circulating adipsin concentrations are increased in prepubertal catch-up SGA children and are associated with features of MASLD. Circulating adipsin may become a novel biomarker of ectopic fat accumulation, linking early growth patterns to markers of metabolic risk.

## 1. Introduction

Metabolic dysfunction-associated steatotic liver disease (MASLD), formerly known as nonalcoholic fatty liver disease (NAFLD), is the most common chronic liver disorder among children and represents the hepatic manifestation of metabolic syndrome [1]. There is growing evidence of fetal programming of MASLD, including the effects of maternal pre- and conceptional health and nutrition, and intrauterine environment [2,3]. In addition, low birth weight as well as rapid postnatal weight gain are also reported to confer an increased risk [4]. MASLD is strongly associated with adipose tissue dysfunction, where hypertrophic adipocytes secrete pro-inflammatory adipokines that trigger systemic inflammation and insulin resistance, ultimately contributing to the development of metabolic syndrome [5].

Adipsin, also known as complement factor-D (CFD), is an adipokine that acts as the rate-limiting factor in the activation of the alternative complement pathway, a major constituent of the innate immunity. Unlike other proteins of the complement system, adipsin is synthesized mainly by adipose tissue, highlighting its important role in regulating adipose tissue function [6]. In this context, it has been shown that adipsin regulates adipocyte differentiation and lipogenesis by inducing peroxisome proliferator-activated receptor gamma (PPARγ) [7]. In addition, adipsin promotes glucose-stimulated insulin secretion by converting complement component C3 into its active form, C3a, which subsequently activates the C3a–C3aR signaling pathway in pancreatic β-cells [8]. Clinical evidence indicates that circulating adipsin concentrations are positively associated with adiposity [9,10], polycystic ovary syndrome (PCOS) [11], fatty liver [12,13], and the development of coronary artery disease [14].

Individuals born small-for-gestational-age (SGA) who experience a rapid and excessive postnatal catch-up in weight are at increased risk for developing obesity, ectopic (hepato-visceral) fat depots, type 2 diabetes (T2D), and cardiovascular disease [15,16,17].

Here, we assessed cross-sectionally for the first time the circulating concentrations of adipsin in a cohort of prepubertal children born either appropriate-for-GA (AGA) or SGA with spontaneous postnatal catch-up and analyzed the associations with auxological and endocrine–metabolic markers, as well as with abdominal fat partitioning and liver fat.

## 2. Results

### 2.1. Anthropometric, Endocrine–Metabolic Variables and Abdominal Fat Partitioning According to Birth Weight

Table 1 shows selected results according to birth weight. SGA children had higher serum adipsin levels as compared to their AGA counterparts (*p* < 0.001), which were not influenced by sex or type of delivery. The SGA subpopulation also displayed a less favorable endocrine–metabolic profile (vs. AGA), including less insulin sensitivity (as evidenced by higher circulating insulin, HOMA-IR, and IGF-1 levels and lower SHBG and HMW-adip concentrations), increased triglycerides, and a higher amount of hepato-visceral fat, in the absence of differences in BMI (vs. AGA).

### 2.2. Bivariate Correlations Between Circulating Adipsin Levels, Auxological, Endocrine–Metabolic Parameters, and Abdominal and Liver Fat

Table 2 shows the associations between circulating adipsin and selected variables. In the entire study population, circulating adipsin levels were inversely associated with birth weight (and its Z-score) and HMW-adip, and positively correlated with BMI (and its Z-score), with the Z-score change from birth weight and BMI at the age of 7 years, with glucose, insulin, HOMA-IR, IGF-1, and triglycerides, and with abdominal (subcutaneous and visceral) fat and liver fat. When the two subgroups were analyzed separately, the aforementioned correlations were only maintained within the SGA subgroup.

### 2.3. Multiple Regression Analysis

Table 3 shows the multivariate regression analyses for liver fat and endocrine–metabolic variables. Adipsin (*p* = 0.005), BMI Z-score (*p* = 0.001), and, to a lesser extent, HOMA-IR (*p* = 0.023) were independently associated with the percentage of liver fat, collectively accounting for nearly 67% of its variance.

### 2.4. Receiver Operating Characteristic (ROC) Curve

**Figure 1:**
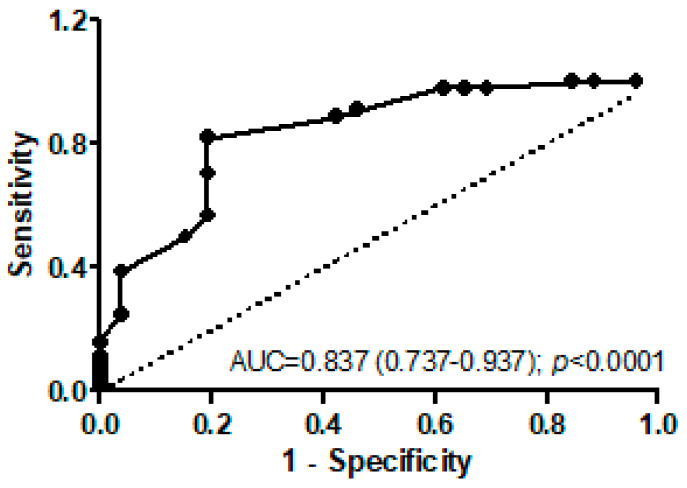
Receiver operating characteristic (ROC) curves depicting the discriminatory effect of circulating adipsin levels to predict liver fat in appropriate- (AGA, n = 40) and small-for-gestational-age (SGA, n = 35) prepubertal children at the age of 7 years.

The ROC curve analysis (Figure 1) disclosed a remarkable capacity of circulating adipsin to predict liver fat [(area under curve (AUC) 0.837 (0.737–0.937), *p* < 0.0001]. According to Youden’s index (sensitivity + specificity − 1), we identified a cut-off value of 1.75 mg/L for adipsin, with a sensitivity and specificity of 82% and 81%, respectively.

**Figure 2:**
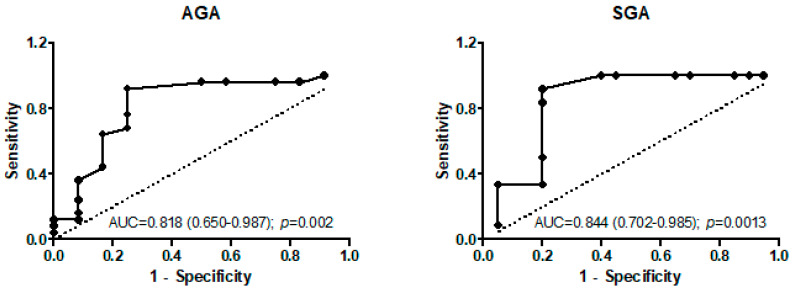
Receiver operating characteristic (ROC) curves depicting the discriminatory effect of circulating adipsin levels to predict liver fat in appropriate- (AGA, n = 40; left panel) or small-for-gestational-age (SGA, n = 35; right panel) prepubertal children at the age of 7 years.

When the analysis was performed separately in the AGA and SGA subgroups, the resulting Youden index (1.75 mg/L) was identical in both groups and comparable to that observed in the combined AGA and SGA analysis (Figure 2). The sensitivity and specificity were 92% and 75% for AGA children, and 92% and 80% for SGA children, respectively.

## 3. Discussion

The present study is the first to report circulating adipsin concentrations in prepubertal AGA and SGA children with catch-up growth without obesity. Serum adipsin levels were significantly higher in SGA children, as compared with those born AGA, and were associated with markers of insulin resistance and ectopic adiposity, both features of MASLD [18]. Furthermore, we disclosed the potential usefulness of adipsin as a biomarker of liver fat content. Collectively, these findings suggest that the alternative complement cascade may play a role in the development of MASLD-like features in SGA subjects.

Several observational studies have reported higher plasma levels of adipsin in subjects with obesity as compared with those without [10,19,20], which decrease after weight loss [20]. The SGA subpopulation included in the present study did not have obesity but had experienced an upward mismatch between prenatal and postnatal weight gain, namely, a situation favoring a hypertrophic rather than a hyperplastic adipose tissue expansion, resulting in ectopic (central) fat accumulation. The higher amount of hepato-visceral fat encountered in SGA children (vs. AGA) supports this notion.

Serum adipsin concentrations were positively correlated with BMI, insulin, IGF-1, and HOMA-IR and negatively correlated with HMW-adip only in SGA subjects. These results are in apparent contrast with studies performed in patients with T2D, disclosing a negative association between adipsin levels and HOMA-IR; alternatively, other studies conducted in similar populations show an increase in adipsin concentrations with increasing severity of obesity [21]. Moreover, higher adipsin concentrations have been reported in PCOS women with insulin resistance as compared with BMI-matched healthy controls [11]. It could thus be speculated that the rise in circulating adipsin levels in SGA children may reflect adipose tissue insulin resistance, leading to altered adipokine secretion and increased lipolysis (as judged by the relatively modest albeit significant elevation in triglycerides), which in turn contributes to further increase in hepatic fat accumulation. Alternatively, the rise in serum adipsin may reflect an adaptive response aimed at enhancing β-cell function to counteract the reduction in insulin sensitivity [8,22].

Serum adipsin levels also showed a positive correlation with subcutaneous, visceral, and liver fat. These results align with the reported association between C3 and adipsin and measures of central adiposity independently of BMI in healthy adolescents [23], suggesting that complement proteins may relate not only to the degree of obesity but also to body fat distribution. They are also consistent with previous findings linking elevated serum adipsin concentrations to an increased risk of MASLD in humans [12] and in high-fat-diet-fed mice, in which adipsin has been shown to stimulate hepatic fatty acid uptake and de novo lipogenesis [24]. Interestingly, human proteomics studies identify serum C3 levels as a key marker for differences in body fat [25,26,27]. The capacity of adipsin to predict the percentage of liver fat in prepubertal children suggests that it may have a potential role as a non-invasive biomarker for the early detection of hepatic steatosis. However, further research involving larger and ethnically diverse pediatric cohorts is required to validate these findings and to establish a reliable and clinically meaningful cut-off value for adipsin that can be applied in clinical practice.

The main limitation of this preliminary study is the lack of age- and BMI-matched SGA children without spontaneous catch-up to dissect the effects of birth weight from those arising from postnatal growth trajectories on adipsin levels. Moreover, the cross-sectional nature of the study precludes ascertaining the prognostic value of adipsin for predicting liver fat accumulation beyond childhood. Finally, the lack of detailed data on dietary intake, physical activity, and family history of metabolic diseases may have acted as a residual confounder. The strengths include the homogeneity of the study population, the strict eligibility criteria avoiding the overlap between AGA and SGA subpopulations, and the co-availability of extensive auxological, endocrine–metabolic, and imaging data. In conclusion, serum adipsin concentrations are increased in prepubertal catch-up SGA children at the age of ~7 yr and are associated with features of MASLD. Circulating adipsin may become a novel biomarker of liver fat accumulation, linking early growth patterns to markers of metabolic risk.

## 4. Materials and Methods

### 4.1. Study Population

The study cohort consisted of seventy-five prepubertal children [age: 7.8 yr; 40 AGA (48% girls) and 35 SGA (51% girls)], who were originally enrolled into two previous longitudinal studies conducted at Hospital Sant Joan de Déu, Barcelona [28,29,30], where the profile of circulating exosomes from birth to the age of 7 years as well as the proteomic and miRNAs profiling of exosomes at birth were assessed (Figure S1, flow chart). Inclusion criteria were maternally uncomplicated, singleton pregnancy with delivery at term (37–42 weeks); birth weight between 2.9 and 3.8 Kg for AGA (range: −1.0 SD and +1.0 SD) and between 1.9 and 2.6 Kg for SGA (≤−2 SD); exclusive breast- or formula-feeding for at least 4 months; spontaneous catch-up in weight and length in SGA (weight and length Z-score > −2.0 by the age of 1 year) [31]; written informed consent. Exclusion criteria were maternal hypertension, preeclampsia, gestational diabetes, alcohol or drug abuse, congenital malformations, or complications at birth (asphyxia, hypoglycemia, infections, neonatal jaundice, birth injuries, or parenteral nutrition).

### 4.2. Assessments

Gestational age was calculated according to the last menses and confirmed by first-trimester ultrasound (6–12 weeks). Weight and length were measured at birth and at the age of 7 years (range 7–8 yr), and body mass index (BMI) was calculated. Weight, length, and BMI Z-scores were derived by using suitable growth charts for the Spanish population [32]. All participants underwent a standardized physical examination to verify their prepubertal status (stage I by Tanner standards). Blood samples were obtained in the morning after an overnight fast at the age of ~7 yr. Samples were stored at −80 °C until analysis.

Serum glucose was measured by the glucose oxidase method. Insulin, insulin-like growth factor 1 (IGF-1), and sex hormone-binding globulin (SHBG) were assessed by immunochemiluminiscence (DPC IMMULITE 2500, Siemens, Erlangen, Germany). Homeostasis model assessment for insulin resistance (HOMA-IR) was calculated as fasting insulin (mU/L) × fasting glucose (mmol/L)/22.5. Lipids, alanine aminotransferase (ALT), and γ-glutamyltransferase (GGT) were assessed by an enzymatic method (Architect c8000 autoanalyzer, Abbott Laboratories, North Chicago, IL, USA). High-molecular-weight adiponectin (HMW-adip) was measured with a specific human enzyme-linked immunosorbent assay kit (R&D Systems, Minneapolis, MN, USA). The intra- and inter-assay coefficients of variation (CVs) were <9%. Adipsin was measured by ELISA using a specific human ELISA kit (R&D Systems, Minneapolis, MN, USA); the intra- and inter-assay CVs were both <10%.

Abdominal fat partitioning (subcutaneous and visceral fat areas) as well as liver fat percentage were assessed at the age of ~7 yr by magnetic resonance imaging (MRI, Signa LX Echo Speed Plus Excite; General Electric, Milwaukee, WI, USA) as previously reported [33].

### 4.3. Statistics and Ethics

Statistical analyses were performed using GraphPad Prism software 8 (La Jolla, CA, USA) and SPSS Statistics 23.0 (IBM Corp., Armonk, NY, USA). Results are expressed as mean ± SEM. All variables were checked for normality using the Kolmogorov–Smirnov test prior to analyses. Comparisons between groups were performed using an unpaired *t*-test or Mann–Whitney U test for non-parametric variables. For comparisons between groups in categorical variables, the Fisher test was used. Pearson correlation and step-wise multiple regression analyses were performed to study the associations between circulating adipsin levels and auxological, endocrine–metabolic, abdominal fat partitioning, and liver fat. Variables were entered into the regression model based on their clinical relevance and statistical significance in the univariate analyses. To assess the potential of adipsin as a predictor of liver fat content, a receiver operating characteristic (ROC) curve analysis was performed. The level of significance was set at *p* < 0.05.

The study was approved by the Institutional Review Board of Barcelona University, Hospital of Sant Joan de Déu (CEIC pic-130-18). All clinical investigations were conducted according to the principles expressed in the Declaration of Helsinki.

## Figures and Tables

**Table 1:** Data from infants born appropriate-for-gestational-age [AGA, (N = 40)] or small-for-GA [SGA, (N = 35)] at the age of 7 years.

	All AGA N = 40	AGA Girls N = 19	AGA Boys N = 21	All SGA N = 35	SGA Girls N = 18	SGA Boys N = 17
**Perinatal**						
Gestational Age (wk)	39.7 ± 0.2	39.7 ± 0.2	39.7 ± 0.3	**38.5 ± 0.3** ^†††^	**38.3 ± 0.4** ^††^	38.8 ± 0.4
Birth Weight (Kg)	3.3 ± 0.1	**3.2 ± 0.1** *	3.4 ± 0.1	**2.2 ± 0.1** ^†††^	**2.2 ± 0.1** ^†††^	2.3 ± 0.1
Birth Weight Z-score	−0.1 ± 0.1	−0.3 ± 0.1	−0.1 ± 0.1	**−2.4 ± 0.1** ^†††^	−2.3 ± 0.1	−2.5 ± 0.1
Sex (% female)	48	--	--	51	--	--
**Anthropometry**						
Age (years)	7.7 ± 0.1	7.8 ± 0.2	7.5 ± 0.2	7.9 ± 0.1	8.0 ± 0.2	7.8 ± 0.2
Weight (Kg)	26.7 ± 1.0	26.3 ± 1.5	27.1 ± 1.3	28.0 ± 1.4	29.8 ± 2.3	26.1 ± 1.5
Weight Z-score	0.1 ± 0.2	−0.1 ± 0.3	0.2 ± 0.3	−0.1 ± 0.2	0.1 ± 0.4	−0.2 ± 0.3
Height (cm)	126.8 ± 1.2	126.7 ± 1.6	126.8 ± 1.8	127.3 ± 1.5	128.6 ± 2.4	125.6 ± 1.7
Height Z-score	0.2 ± 0.2	0.2 ± 0.3	0.2 ± 0.3	−0.2 ± 0.2	−0.3 ± 0.3	−0.1 ± 0.3
BMI Z-score	−0.1 ± 0.2	−0.3 ± 0.3	0.1 ± 0.2	−0.1 ± 0.2	0.1 ± 0.3	−0.2 ± 0.2
Δ BW-BMI Z-score	0.1 ± 0.2	0.1 ± 0.2	0.1 ± 0.2	**2.4 ± 0.2** ^†††^	2.5 ± 0.3	2.3 ± 0.3
**Endocrine–metabolic variables**						
Glucose (mmol/L)	4.7 ± 0.1	4.6 ± 0.1	4.9 ± 0.1	4.8 ± 0.1	4.8 ± 0.1	4.8 ± 0.1
Insulin (pmol/L)	20 ± 2	17 ± 3	22 ± 3	**38 ± 5** ^††^	40 ± 6	28 ± 5
HOMA-IR	0.6 ± 0.1	0.5 ± 0.1	0.6 ± 0.1	**1.2 ± 0.2** ^††^	1.2 ± 0.2	0.9 ± 0.2
IGF-1 (nmol/L)	20 ± 1	20 ± 1	19 ± 1	**28 ± 2** ^††^	30 ± 2	24 ± 2
SHBG (nmol/L)	110 ± 6	100 ± 8	120 ± 9	**90 ± 6** ^†^	81 ± 10	101 ± 6
Triglycerides (mmol/L)	0.54 ± 0.03	0.55 ± 0.04	0.53 ± 0.04	**0.63 ± 0.03** ^†^	0.64 ± 0.03	0.62 ± 0.05
HDL Cholesterol (mmol/L)	1.65 ± 0.05	1.58 ± 0.07	1.71 ± 0.08	**1.47 ± 0.06** ^†^	1.41 ± 0.08	1.54 ± 0.08
LDL Cholesterol (mmol/L)	2.38 ± 0.11	2.52 ± 0.14	2.25 ± 0.16	2.19 ± 0.08	2.08 ± 0.11	2.32 ± 0.13
ALT (IU/L)	16 ± 1	17 ± 1	15 ± 1	14 ± 1	14 ± 1	14 ± 1
GGT (IU/L)	12 ± 1	11 ± 1	12 ± 1	12 ± 1	12 ± 1	12 ± 1
HMW-adip (mg/L)	13.5 ± 1.1	13.0 ± 1.8	13.9 ± 1.4	**9.1 ± 1.2** ^††^	9.3 ± 2.2	8.7 ± 4.2
Adipsin (mg/L)	1.4 ± 0.1	1.3 ± 0.1	1.5 ± 0.1	**1.9 ± 0.1** ^†††^	2.0 ± 0.2	1.8 ± 0.1
**Abdominal fat partitioning (MRI)**						
Subcutaneous fat (cm^2^)	40 ± 4	36 ± 4	36 ± 5	53 ± 7	50 ± 9	37 ± 6
Visceral fat (cm^2^)	15 ± 1	18 ± 2	17 ± 1	**19 ± 1** ^†^	20 ± 2	17 ± 2
Liver fat (%)	11 ± 1	12 ± 1	10 ± 1	**15 ± 1** ^††^	15 ± 1	15 ± 1

Values are mean ± SEM. BMI, body mass index; BW, birth weight; HOMA-IR, homeostasis model assessment of insulin resistance; IGF-1, insulin-like growth factor 1; SHBG, sex hormone-binding globulin; HDL, high-density lipoprotein; LDL, low-density lipoprotein; ALT, alanine aminotransferase; GGT, gamma-glutamyl transferase; HMW-adip, high-molecular-weight adiponectin. Numbers in bold highlight significant differences. ^†^ *p* < 0.05, ^††^ *p* < 0.01, ^†††^ *p* < 0.001 vs. AGA. * *p* < 0.05 vs. boys.

**Table 2:** Bivariate correlations between circulating adipsin levels and selected parameters from 7-yr-old children born appropriate- [AGA, N = 40] or small-for-gestational-age [SGA, N = 35].

	Adipsin (mg/L)									
	All		AGA		SGA		SGA Girls		SGA Boys	
	R	P	r	P	r	P	R	P	r	P
**Anthropometry**										
BMI SDS at the age of 7 years	0.315	0.008	--	Ns	0.460	0.009	0.689	0.006	0.673	0.033
Δ BW-BMI Z-score	0.386	0.0007	--	Ns	0.423	0.018	0.657	0.015	0.625	0.022
**Endocrine–metabolic variables**										
HOMA-IR	0.321	0.007	--	Ns	0.431	0.022	0.695	0.012	0.652	0.029
HMW-adip	−0.404	0.0004	--	Ns	−0.447	0.008	−0.643	0.034	−0.554	0.032
**Abdominal MRI**										
Subcutaneous fat	0.351	0.003	--	Ns	0.492	0.004	0.727	0.005	0.598	0.024
Visceral fat	0.336	0.006	--	Ns	0.439	0.013	0.703	0.007	0.652	0.011
Liver fat	0.335	0.006	--	Ns	0.412	0.021	0.745	0.005	0.712	0.031

BMI, body mass index; BW, birth weight; HOMA-IR, homeostasis model assessment of insulin resistance; HMW-adip, high-molecular-weight adiponectin.

**Table 3:** Multivariate linear model of liver fat and selected endocrine–metabolic parameters at the age of 7 years.

	Liver Fat at the Age of 7 Years		
	Beta	Sig.	R^2^
**Adipsin at the age of 7 years**	0.485	0.005	0.263
**BMI Z-score at the age of 7 years**	0.337	0.001	0.301
**HOMA-IR at the age of 7 years**	0.251	0.023	0.105

BMI, body mass index; HOMA-IR, homeostatic model insulin resistance. Non-predictive variables: subcutaneous fat, visceral fat, high-molecular-weight-adiponectin, insulin-like growth factor-1, triglycerides, and high-density lipoprotein cholesterol at the age of 7 years.

## Data Availability

The original contributions presented in this study are included in the article/Supplementary Material. Further inquiries can be directed to the corresponding author.

## Supplementary Materials

The following supporting information can be downloaded at: https://www.mdpi.com/article/10.3390/ijms27115023/s1.
