# Supplementary material for: Circulating Adipsin as a Biomarker of Liver Fat Content in Prepubertal Children Born Small-for-Gestational-Age
Source: Int J Mol Sci. 2026 Jun 2;27(11):5023. doi: 10.3390/ijms27115023 (PMC13256761; doi:10.3390/ijms27115023)
Supplement: Supplementary file 1 [file ijms-27-05023-s001.zip › ijms-4287496-supplementary.pptx]

## Slide 1
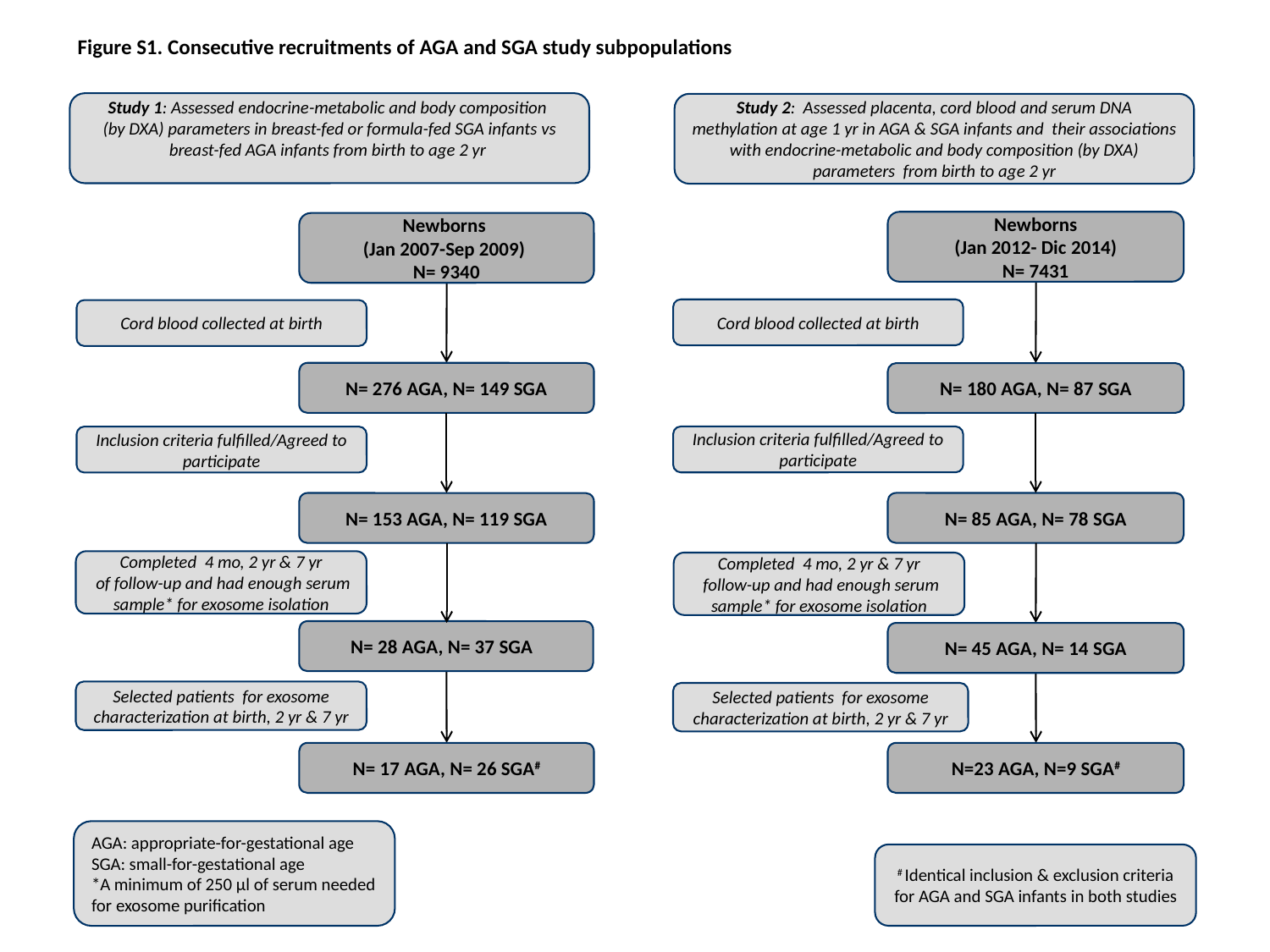

Figure S1. Consecutive recruitments of AGA and SGA study subpopulations
Study 1: Assessed endocrine-metabolic and body composition
 (by DXA) parameters in breast-fed or formula-fed SGA infants vs  breast-fed AGA infants from birth to age 2 yr
Study 2: Assessed placenta, cord blood and serum DNA methylation at age 1 yr in AGA & SGA infants and their associations with endocrine-metabolic and body composition (by DXA) parameters  from birth to age 2 yr
Newborns
(Jan 2012- Dic 2014)
N= 7431
Newborns
(Jan 2007-Sep 2009)
N= 9340
N= 276 AGA, N= 149 SGA
N= 180 AGA, N= 87 SGA
N= 85 AGA, N= 78 SGA
N= 153 AGA, N= 119 SGA
N= 28 AGA, N= 37 SGA
N= 45 AGA, N= 14 SGA
N= 17 AGA, N= 26 SGA#
N=23 AGA, N=9 SGA#
Cord blood collected at birth
Cord blood collected at birth
Inclusion criteria fulfilled/Agreed to
participate
Inclusion criteria fulfilled/Agreed to
participate
Completed 4 mo, 2 yr & 7 yr
 of follow-up and had enough serum sample* for exosome isolation
Completed 4 mo, 2 yr & 7 yr
 follow-up and had enough serum sample* for exosome isolation
Selected patients for exosome characterization at birth, 2 yr & 7 yr
Selected patients for exosome characterization at birth, 2 yr & 7 yr
AGA: appropriate-for-gestational age
SGA: small-for-gestational age
*A minimum of 250 µl of serum needed for exosome purification
# Identical inclusion & exclusion criteria for AGA and SGA infants in both studies
